# An RNA Polymerase III-Dependent Heterochromatin Barrier at Fission Yeast Centromere 1

**DOI:** 10.1371/journal.pone.0001099

**Published:** 2007-10-31

**Authors:** Kristin C. Scott, Caroline V. White, Huntington F. Willard

**Affiliations:** Institute for Genome Sciences and Policy, Duke University, Durham, North Carolina, United States of America; Texas A&M University, United States of America

## Abstract

Heterochromatin formation involves the nucleation and spreading of structural and epigenetic features along the chromatin fiber. Chromatin barriers and associated proteins counteract the spreading of heterochromatin, thereby restricting it to specific regions of the genome. We have performed gene expression studies and chromatin immunoprecipitation on strains in which native centromere sequences have been mutated to study the mechanism by which a tRNA^Alanine^ gene barrier (cen1 tDNA^Ala^) blocks the spread of pericentromeric heterochromatin at the centromere of chromosome 1 (cen1) in the fission yeast, *Schizosaccharomyces pombe*. Within the centromere, barrier activity is a general property of tDNAs and, unlike previously characterized barriers, requires the association of both transcription factor IIIC and RNA Polymerase III. Although the cen1 tDNA^Ala^ gene is actively transcribed, barrier activity is independent of transcriptional orientation. These findings provide experimental evidence for the involvement of a fully assembled RNA polymerase III transcription complex in defining independent structural and functional domains at a eukaryotic centromere.

## Introduction

Eukaryotic genomes are packaged into two main categories of chromatin that can determine the behavior of the underlying DNA sequence. Euchromatin is typically found in gene rich regions of the genome that are accessible to factors involved in various biological processes including transcription, replication and recombination. In contrast, regions of heterochromatin are generally gene poor, and confer transcriptional repression to inserted reporter genes [1]. Heterochromatin also is required for proper chromosome segregation [2], [3]. Hypoacetylation of histones and methylation of histone H3 at lysine 9 are distinguishing marks of heterochromatin in many eukaryotic genomes, including the fission yeast *Schizosaccharomyces pombe (S. pombe)* [4]. In addition to the general categories of heterochromatin and euchromatin, a third type of specialized chromatin exists at centromeres. Centromeric chromatin contains blocks of canonical nucleosomes, methylated on H3 lysine 4, interspersed with blocks of nucleosomes containing the histone H3 variant, cenH3, a protein that provides the structural and functional foundation of all active kinetochores [5].

A key feature of heterochromatin is its ability to spread in *cis*, causing epigenetic silencing of an otherwise euchromatic gene [6]. The genomic and/or epigenetic features at the confluence of discrete chromatin domains remain poorly understood; however, at some loci a specialized class of DNA element, known as a chromatin barrier [7], plays an active role in demarcating the different chromatin states. Chromatin barriers restrict heterochromatin to specific genomic regions and fall within a broader class of elements called insulators [8]. The range of proteins associated with chromatin barriers is incompletely defined, although increasing evidence suggests that barrier activity correlates with the recruitment of histone acetylase activity and/or the assembly of a transcription complex [9], [10], [11], [12]. Accordingly, many insulators are coincident with the promoters of genes [13], [14], [15]

We recently defined a novel chromatin barrier element in the fission yeast genome that partitions centromere 1 (cen1) chromatin into structurally distinct domains of pericentromeric heterochromatin and centromeric chromatin [16]. The absence of this barrier results in both propagation of pericentromeric heterochromatin beyond its normal boundary into centromeric chromatin, as well as defects in chromosome segregation during meiosis. Barrier activity is dependent upon an intact transfer RNA alanine gene (cen1 tDNA^Ala^) that is transcribed from its endogenous, centromeric location [16]. In this study, we further characterize the properties of this novel cen1 tDNA^Ala ^barrier, which differ in several aspects from previously described fission yeast barriers.

## Results

### Barrier activity is a general feature of centromeric tDNAs

**Figure 1:**
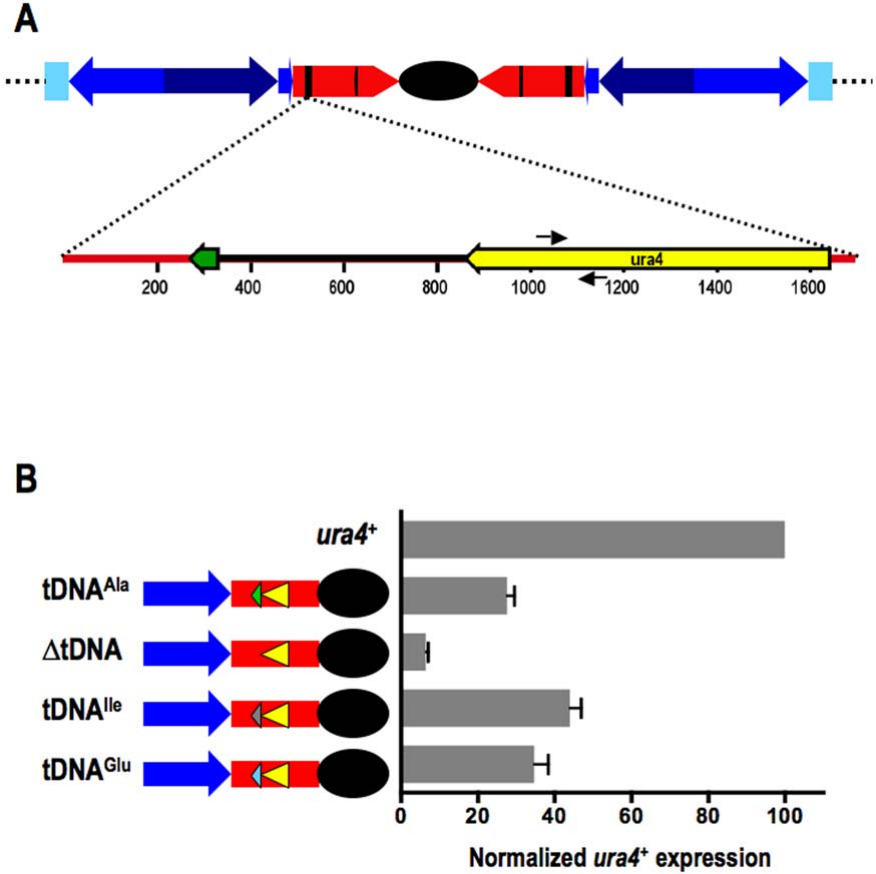
Barrier activity is a general property of tDNAs . A. (Upper) Representation of *S. pombe* centromere 1 (cen1). The central core (black) is surrounded by inverted repeats including the inner repeat (imr1; red) and outer repeats (otr1-; light blue, navy and purple). Black vertical lines within imr represent tDNAs in cen1. (Lower) Map of cen1 tDNA^Ala^ (green) and surrounding sequence. The *ura4^+^*reporter gene (ORF,yellow arrow; surrounding sequence, black line) was inserted into imr1 of cen1. PCR primers are shown as black arrows. B. Illustrations to the left of the graph as above. tDNA alanine (green), isoleucine (gray) and glutamine (light blue) are represented as colored triangles in imr1. Real time RT-PCR analysis of centromeric *ura4^+^* transcription was normalized to endogenous *ura4^+^*transcription and compared among indicated strains. Error bars represent the standard error of the mean (SEM).

tDNAs in the fission yeast genome range in size from 71–102 bp and are characterized by highly conserved internal control elements. To determine whether barrier activity is a general property of tDNAs or if the cen1 tDNA^Ala^ has additional sequence features that convey barrier activity, we engineered strains in which cen1 tDNA^Ala^ (including 40 bp upstream sequence and 25 bp downstream) was replaced with tDNAs that encode two different fission yeast tRNA isotypes . The presence of cen1 tDNA^Ala^ blocks the spread of heterochromatin and permits gene expression from a centromere proximal *ura4^+^*reporter gene (at a moderate level of 27% of wildtype *ura4^+^*activity), as observed previously ([16]; Fig 1). In the absence of these sequences, however, the spread of pericentromeric heterochromatin results in a significant reduction of *ura4^+^*reporter gene expression to only ∼6% of wild-type levels. In comparison, replacement of cen1 tDNA^Ala^ with non-centromeric tDNA^Glu^ or tDNA^Ile^ restored reporter gene expression (35% and 44%, respectively), suggesting that the ability to block the spread of heterochromatin at the centromere is a general property of tDNAs in fission yeast.

### RNA polymerase III and TFIIIC associate with the tDNA barrier

Transcription of tDNAs by RNA polymerase III (Pol III) involves the multi-step assembly of transcription factors into a pre-initiation complex that recruits Pol III [17] . Two highly conserved internal control regions, the A and B boxes, together form a specific binding site for the multisubunit transcription factor IIIC (TFIIIC). Promoter bound TFIIIC provides an interaction platform for the productive assembly of TFIIIB at a TATA box immediately upstream of tDNAs [17], [18]. TFIIIB next recruits Pol III and transcription proceeds through a facilitated re-initiation pathway that involves polymerase recapture after transcription termination [19]. Previous work has indicated that cen1 tDNA^Ala^ and its surrounding sequences are sensitive to micrococcal nuclease digestion [20] and correspond to DNAse I hypersensitive sites [21]. Furthermore, as we have shown previously, cen1 tDNA^Ala^ is transcribed and both intact TATA and A box regions are required for robust barrier activity [16]. These findings together suggest that Pol III and its associated transcription complex gain access to the tDNA barrier at the centromere, thus forming a large stable DNA-bound complex.

**Figure 2:**
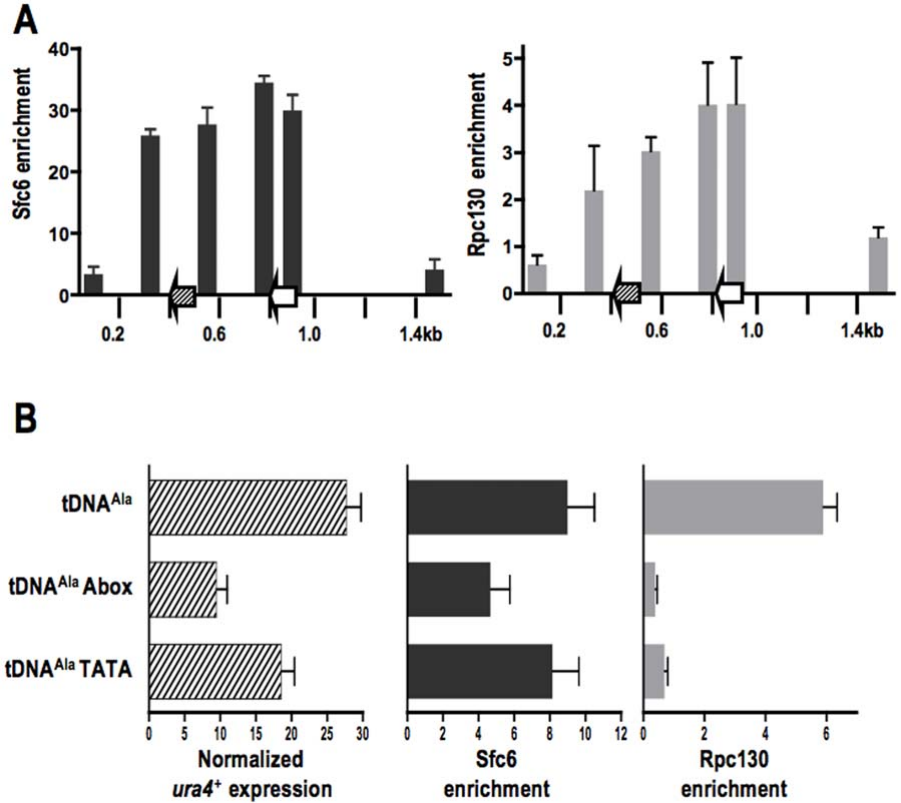
Barrier activity requires the RNA polymerase III complex. A. Chromatin IP analysis of cen1 for enrichment of Sfc6 (TFIIIC) and Rpc130 (Pol III). The X-axis represents 1.5 kb of cen1 imr including tDNA^Ala^ (green) and nearby tDNA^Glu^ (white). Centromere specific primers are listed in Materials and Methods. Error bars represent SEM. B . Real time RT-PCR analysis of centromeric *ura4^+^* transcription normalized to endogenous *ura4^+^* transcription in strains containing wild-type or mutant barriers (Left). Indicated strains were analyzed by chromatin IP for Sfc6 (Middle, dark gray) and Rpc130 (Right, light gray) enrichment. Error bars represent SEM.

Genome-wide chromatin immunoprecipitation (ChIP on microarray) previously demonstrated low, but measurable levels of association of both TFIIIC and Pol III with cen1 tDNAs [22]. We confirmed this observation by ChIP analysis over a 1.6 kb region surrounding tDNA^Ala^. These studies revealed that both Sfc6, a TFIIIC subunit, and Rpc130, a Pol III subunit [17], were enriched at cen1 tDNA^Ala^ 28- and 3- fold, respectively, over a non-centromeric control locus (Figure 2A).

We next investigated whether components of TFIIIC and Pol III are present at centromeres where barrier activity has been compromised by mutation of either the A box internal control element or the upstream TATA box (Figure 2B, [16]). Using specific primers to distinguish the modified barrier, which also bears the *ura4^+^* reporter gene, from the wild-type tDNA^Ala^ on the inverted repeat of cen1, we confirmed that both Sfc6 and Rpc130 were present at this locus. However, association of Rpc130 was essentially abolished in strains carrying the mutant barrier (Figure 2B, right panel). Enrichment of Sfc6 was also investigated in these strains. The A box mutation displayed ∼49% reduction in Sfc6, whereas the level of enrichment in TATA box mutants was not changed (Figure 2B, middle panel). Importantly, both mutations have reduced levels of *ura4^+^* expression (9.4% and 18.5% of wildtype *ura4^+^* expression, respectively; Figure 2C left panel), and, therefore, reduced barrier activity. Taken together, these data demonstrate that Sfc6 association is not sufficient to counter the spread of pericentromeric heterochromatin [22] and suggest that robust cen1 tDNA^Ala^ barrier activity requires the assembly of a full Pol III transcription complex.

### Barrier activity is independent of tDNA orientation

Transcriptional interference between Pol III and RNA polymerase II (Pol II) genes has been described in *Saccharomyces cerevisiae*. This tDNA “position effect” has been observed mostly with selected artificial constructions, in which Pol II-transcribed reporter genes were found to be inhibited 2-to 60- fold by a neighboring tDNA [23], [24]. However modest tDNA position effects have also been reported to operate at native chromosomal loci, particularly when the Pol II-transcribed gene is less than 1000 bp from a tDNA [25], [26], [27].

Assembly of pericentromeric heterochromatin in fission yeast requires Pol II transcription of the outer repeats [28], followed by processing of the transcripts by the RNAi machinery [1]. Compellingly, siRNAs within 1kb of tDNA^Ala^ have been identified [29], although due to both the small size of siRNAs and the sequence redundancy of fission yeast centromeres, their origin cannot be confirmed. These observations suggest the possibility that barrier activity results from a position effect that represses Pol II transcription of repetitive DNA, thus weakening local pericentromeric heterochromatin assembly at tDNA^Ala^. Thus, we hypothesized that barrier activity may be dependent upon the orientation of transcription of tDNA^Ala^. As a control, we first determined whether *ura4^+^*reporter gene expression is influenced by cen1 tDNA^Ala^ in the absence of heterochromatin. In strains that lack Clr4, the histone methyltransferase [30] required for pericentromeric heterochromatin assembly[31], [32], expression of the centromeric *ura4^+^*reporter gene is ∼95% (+/−6%) of chromosomal *ura4^+^*, demonstrating that the Pol III complex has no significant positive or negative position effects on the *ura4^+^*reporter gene when in a euchromatic environment. Next, we engineered strains in which the direction of both cen1 tDNA^Ala^ and *ura4^+^*transcription was reversed, and assayed reporter gene expression in these otherwise wild-type strains. Our results indicate that barrier activity is independent of tDNA transcriptional orientation (Table 1).

**Table 1:** Barrier activity is independent of tDNA orientation

Genotype^a^	tDNA^Ala ^ b	ura4^+^ c	Normalized ura4^+^ expression % d	S.E.M%^e^
Cen1::ura4^+^ clr4::Leu2	N	F, R	94.8	5.6
Cen1::ura4^+ ^clr4^+^	N	R	30.1	4.7
Cen1::ura4^+ ^clr4^+^	N	F	25.2	3.9
Cen1::ura4^+ ^clr4^+^	R	R	26.4	4.0
Cen1::ura4^+ ^clr4^+^	R	F	31.9	5.2

a Strains contained the ura4^+^ reporter gene at centromere 1 in both wild type and clr4^−^ backgrounds.

b Transcriptional orientation of tDNA^Ala^: N, native (antisense strand); R, reverse (sense strand).

c Transcriptional orientation of the ura4^+^ reporter gene: F, forward (sense strand); R, Reverse (antisense strand).

d ura4^+^ transcript levels were measured as in Figure 1.

e standard error of the mean

## Discussion

**Figure 3:**
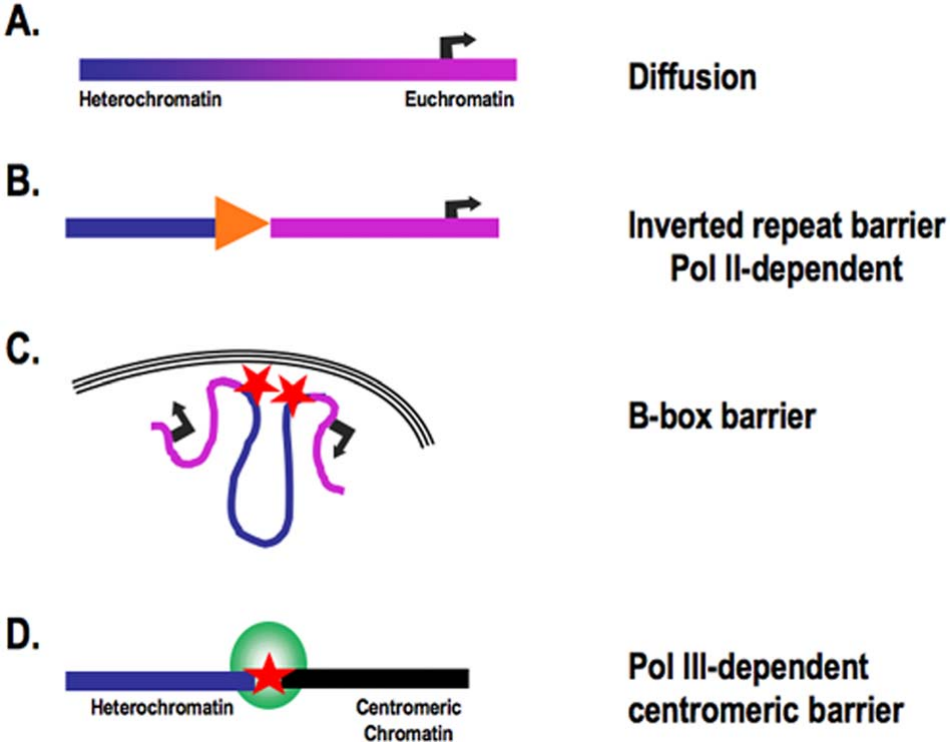
Types of barriers in fission yeast. A. Boundaries of the distinct chromatin domains (dark blue, heterochromatin; pink, euchromatin) are fluid and established through counteracting processes dependent upon the local concentrations of activators and repressors. B. Inverted Repeat barriers are transcribed by RNA pol II (orange arrow) and associated with active chromatin modifications. C. B-box barriers associate with TFIIIC (red star) and coalesce at the nuclear periphery. D. cen tDNA barriers distinguish pericentromeric heterochromatin from centromeric chromatin and require association of both TFIIIC and RNA Pol III (green circle). See discussion for details.

Transitions between discrete chromatin types have been studied in many organisms, from yeast to vertebrates[8], [33]. In fission yeast, three structurally and genomically distinct types of boundaries have been described (Figure 3A–C). At telomeres, chromatin domains are fluid and broad and are believed to depend on the balance between opposing effects of histone modifying activities and/or histone variants[29], [34], rather than on specific genomic sequences that serve as chromatin boundary elements. A second type of boundary is defined by inverted repeats at the centromere. This class of barrier is transcribed by Pol II [29] and, like the vertebrate 5′HS4 β- globin barrier [35], is enriched in active chromatin modifications such as histone acetylation and H3K4me [36]. These euchromatic modifications may act as a chain terminator for the propagation process that generates methylated pericentromeric heterochromatin [12], [37]. A third type of boundary flanks the silent mating type cassettes. These inverted repeats contain several Pol III B Box motifs, and barrier activity requires the association of TFIIIC. It is important to note that B box barriers do not associate with Pol III and have been proposed to function by forming chromatin loops and/or by altering the nuclear localization of domains of chromatin, since dispersed sites of TFIIIC association coalesce at the nuclear periphery [22].

Here, we demonstrate a fourth type of barrier in fission yeast (Figure 3D), using strains in which native tDNA barrier sequences at cen1 have been altered. Centromeric tDNA barriers require the assembly of a fully functional Pol III promoter complex. This is exemplified most strongly by strains in which the A box internal control element has been mutated (Figure 2). In this case, Sfc6 (TFIIIC) is recruited, presumably through the unaltered B box sequences; however, it is not sufficient to counter the spread of pericentromeric heterochromatin. Barrier activity is observed only when the Pol III complex is recruited to the centromere. Thus, centromeric tDNA barriers are mechanistically distinct from previously described heterochromatin barriers in fission yeast.

Pol III is a nuclear enzyme that has been specialized to produce small non-translated RNAs in great abundance [38]. While our studies demonstrate that barrier activity is independent of the orientation of tDNA transcription, they do not address whether transcription is required for barrier activity or whether complex formation, in the absence of transcription, is sufficient. Mutations that impair the enzymatic activity of the Pol III complex, but not its assembly, have not been identified, and the development of such mutations represents an important area of future work. The fully assembled Pol III complex is at least 1.3 MDa [38], footprints nearly 150 bp along the chromatin fiber [39], [40], preventing nucleosome placement [41], [42], [43]. Based on the data presented here, we hypothesize that the Pol III complex at cen1 likely behaves as a chain terminator (and hence a barrier) to the nucleosome-dependent propagation of pericentromeric heterochromatin.

Cen1 tDNA^Ala^ barrier activity may also result from the cooperative effects of more than one mechanism [12], [37]. Interestingly, two histone demethylases (*lsd1^+^* and *lsd2^+^*) associate with cen1 tDNA^Ala^, and the deletion of *lsd1* results in the propagation of pericentromeric heterochromatin beyond tDNA^Ala^ [44], suggesting that histone demethylases contribute to barrier activity by locally regulating histone methylation levels. Moreover, transcriptionally active tDNAs in *S. cerevisiae* localize to the nucleolus[45], [46], raising the possibility that barrier activity correlates with nuclear position.

tDNA barriers have also been demonstrated in the budding yeast *S. cerevisiae*, where silencing is mediated by Sir proteins. However, not all budding yeast tDNAs are capable of barrier activity [47], due to differing affinities of TFIIIB for tDNA upstream sequences, the association of the HMG-like protein Nhp6 [48], as well as the distance between the internal control elements [47]. In contrast, we have shown here that other fission yeast tDNA sequences do retain barrier activity in our centromere specific assay (Figure 1). Notably, Pol III transcription complex assembly differs significantly between the budding and fission yeasts: fission yeast tDNAs have an upstream TATA element, centered at position −30 that participates in direct recruitment of TFIIIB [18]. Indeed, TATA motifs can be identified at all three tDNAs shown to have barrier activity in this study. Intriguingly, however, a significant difference in the strength of barrier activity was observed between strains containing tDNA^Ala ^and tDNA^Ile ^(p<0.001) (Fig 1). We suggest that more potent barrier activity may arise either as a consequence of variable TFIIIB affinity for different TATA boxes or because of a 23 bp increase in the tDNA^Ile^ constructs between heterochromatin nucleating DNA at cen1 and the *ura4^+^* reporter gene. It is noteworthy that a single tDNA flanking the left side of cen1, between pericentromeric heterochromatin and euchromatin, lacks barrier activity, despite its association with both TFIIIC and Pol III [22], suggesting that the centromere provides a favorable environment for tDNA barrier activity.

The domain organization at each of the three fission yeast centromeres is similar and involves transitions among three types of chromatin: heterochromatin, euchromatin and centromeric chromatin. Previous genome-wide mapping of covalently modified histones showed an abrupt transition from marks associated with pericentromeric heterochromatin to those associated with centromeric chromatin [29]. Both chromatin types are essential for centromere activity [49], and mis-segregation events are observed in the absence [50], [51] or mislocalization [16] of heterochromatin. Compellingly, each chromatin transition correlates with the presence of a pair or cluster of tDNAs [29], including seven different tRNA isotypes [20]. We speculated previously that the neighboring tDNAs perform an essential barrier function at fission yeast centromeres, supported by our observation of chromsosome missegregation during meiosis in the absence of tDNA^Ala^ [16]. Thus, unlike conventional barriers that ensure appropriate euchromatic gene expression in euchromatin (Figure 3; A–C), cen tDNA barriers may ensure proper centromere assembly and function by protecting the central core domain of centromeric chromatin from the distinct structural changes and epigenetic features associated with pericentromeric heterochromatin.

## Materials and Methods

### Plasmid DNAs

Plasmids SM353 and SM349 [16] contain 1.78 kb of imr1 sequence, including the cen1 tDNA^Ala^ and a *ura4^+^* reporter gene at the unique HindIII site. These plasmids were modified by site directed mutagenesis to introduce unique ClaI (−45) and EcoRI(+20) sites flanking tDNA^Ala^. tDNA^Glu ^, tDNA^Ile^, and reverse tDNA^Ala^ were amplified from genomic DNA with primers containing either EcoRI or ClaI restriction sites. The resulting PCR products were purified, digested with EcoRI and ClaI, and ligated with vector that had been digested with EcoRI and ClaI to liberate tDNA^Ala^. Resulting plasmids were purified, sequenced and digested with Not1 and KpnI to generate a 3.5 kb product used for yeast transformation.

### Fission yeast strains

The genotypes for *S. pombe* strains used in this study are listed in Table 2. Media were prepared according to standard procedures [52]. All transformations were performed as in [16]. At least three independent transformed strains were established from each construct. Strains were crossed 2–3 times to KFY 3/4 before further analyses.

**Table 2:** Strains used in this study

2	h- ade6-210
3	h+ ura4D18 ade6-210 leu1-32 his3D arg3D4
4	h- ura4D18 ade6-210 leu1-32 his3D arg3D4
104	h90 swi6::arg ura4D18 leu1-32 ade6-210 his3D arg3D4
139	h+ imr1L (Δala HindIII)::ura4 ura4D18 ade6-210 leu1-32 his3D arg3D4
146	h+ imr1R (Δala HindIII)::ura4 ura4D18 ade6-210 leu1-32 his3D arg3D4
188	h+ imr1L(Hind III)::ura4 leu1-32 ade6-210 ura4D18 arg3D4
199	h90 imrL(HindIII)::ura4 clr4::leu2 ade6-210 ura4D18 arg3D4 his3D
201	h90 imrR(HindIII)::ura4 clr4::leu2 ade6-210 ura4D18 arg3D4 his3D
205	h+ imrR(HindIII)::ura4 arg3D4 leu1-32 ade6-210 ura4D18 his3D
258	h- imr1L (Ala TATA* HindIII)::ura4 arg3D4 his3D leu1-32 ura4D18 ade6-210
262	h- imr1R (Ala Abox* HindIII)::ura4 arg3D4 his3D leu1-32 ura4D18 ade6-210
263	h- imr1R (Ala Abox* HindIII)::ura4 arg3D4 his3D leu1-32 ura4D18 ade6-210
301	h? imr1L (Ala TATA* HindIII)::ura4 arg3D4 his3D leu1-32 ura4D18 ade6-210
302	h? imr1L (Ala TATA* HindIII)::ura4 arg3D4 his3D leu1-32 ura4D18 ade6-210
354	h? imr1R (Ala Abox* HindIII)::ura4 arg3D4 his3D leu1-32 ura4D18 ade6-210
504	h? imr1L (glu-glu HindIII)::ura4 arg3D4 his3D leu1-32 ura4D18 ade6-210
506	h? imr1L (glu-glu HindIII)::ura4 arg3D4 his3D leu1-32 ura4D18 ade6-210
535	h+ imr1R(ile-glu HindIII)::ura4 arg3D4 his3D leu1-32 ura4D18 ade6-210
699	h? imr1R(glu-glu HindIII)::ura4 arg3D his3D leu1-32 ura4 D18 ade6-210
700	h? imr1R(glu-glu HindIII)::ura4 arg3D his3D leu1-32 ura4 D18 ade6-210
772	h? imr1L(ala reverse HindIII)::ura4 ade6-210 leu1-32 ura4D18 his3D arg3D4
773	h? imr1L(ala reverse HindIII)::ura4 ade6-210 leu1-32 ura4D18 his3D arg3D4
774	h? imr1L(ala reverse HindIII)::ura4 ade6-210 leu1-32 ura4D18 his3D arg3D4
1024	h- imr1R(ile-glu HindIII)::ura4 arg3D4 his3D leu1-32 ura4D18 ade6-210
1025	h+ imr1R(ile-glu HindIII)::ura4 arg3D4 his3D leu1-32 ura4D18 ade6-210

### Real-Time RT-PCR

Yeast were grown in YES to 5×10^6^ cells/ml at 32°C. cDNA was prepared by oligo dT primed RT-PCR (Invitrogen). Real-time PCR was performed in the presence of SYBR Green on a Bio-Rad iCycler with the following primer pairs for *ura4^+^*: 5026, 5′-TGATATGACCCCAAGAAGCA-3′; 5027, 5′-AAAAACTGGTGGCCTTAGGT-3′ and *act1^+^*: 7453, 5′- AATCCAACCGTGAGAAGATGA-3′; 7454, 5′- ACGACCAGAGGCATACAAAGA-3′. A standard curve of at least four orders of magnitude was generated with genomic DNA isolated from the wild-type strain (*ura4^+^* KFY2). Data were analyzed with iCycler iQ Optical System Software. Data were analyzed further only if the PCR efficiency was between 90–110% and the correlation coefficient was between 0.990 and 1.0. *ura4^+^* levels were normalized to *act1^+^* levels and quantified relative to the wild-type strain. At least three biological replicates were peformed for each mutant.

## ChIP

∼2.5×10^8^ cells were grown to mid-log phase at 32°C, then shifted to 18°C for two hours. Cells were pelleted, fixed in 3% paraformaldehyde for 30 min, pelleted and washed twice in ice-cold PBS. Cells were resuspended in 10 ml of 10 mM dimethyladipidate, including 0.0025% DMSO and incubated at room temperature for 30 min. Cells were pelleted and washed twice in ice cold PBS. ChIP was performed as previously described [16]. 7ul of anti-Sfc6 or anti-Rpc130 antisera [18]; [17] were used for each ChIP. PCR products were quantified as described for RT-PCR. Enrichment at each query locus was calculated relative to the euchromatic *act1^+^* value and then corrected for the ratio obtained for the input PCR. Centromeric primers: 5373, 5′-TCATTCGTTGTACCAACTGCT-3′; 5374, 5′- AAACACCATGGTTTGTTTGTTA-3′; 5375, 5′-TCATTCGTTGTACCAACTGCT-3′; 5376, 5′-TGTGTTTGCCATCTTACAATTCA-3′; 5520, 5′- CACCACATGCCCTAATTGTT-3′; 5521, 5′-TGCGTTCATCTAAAAGCTTCA-3′; 5562, 5′-CGCTACTCATCTGTTTCGTGT-3′; 5563, 5′-CCCCTGACGGAGAAGTTTTAT-3′, 5522, 5′- CCATGACGGATGCTTAGTTCA-3′; 5523, 5′-TAAATTATCGCAGCCTTTCAA-3′; 5564, 5′- GCGAAAACTTTTGATGGAGAG-3′; 5565, 5′-GGTTTTGGTTTTTCTTCCCAG-3′; ura4/cen primers: 5590, 5′-GCCCTAATTGTTTATTTTAGCG-3′; 5591, 5′-CAAAGCCAATGAAAGATGTATGTAG-3′. At least three independent ChIP experiments were performed with each antisera.

## References

[1] Huisinga KL, Brower-Toland B, Elgin SC (2006). The contradictory definitions of heterochromatin: transcription and silencing.. Chromosoma.

[2] Jia S, Yamada T, Grewal SI (2004). Heterochromatin regulates cell type-specific long-range chromatin interactions essential for directed recombination.. Cell.

[3] Pidoux A, Mellone B, Allshire R (2004). Analysis of chromatin in fission yeast.. Methods.

[4] Grewal SI, Elgin SC (2002). Heterochromatin: new possibilities for the inheritance of structure.. Curr Opin Genet Dev.

[5] Sullivan BA, Karpen GH (2004). Centromeric chromatin exhibits a histone modification pattern that is distinct from both euchromatin and heterochromatin.. Nat Struct Mol Biol.

[6] Talbert PB, Henikoff S (2006). Spreading of silent chromatin: inaction at a distance.. Nat Rev Genet.

[7] Sun FL, Elgin SC (1999). Putting boundaries on silence.. Cell.

[8] Valenzuela L, Kamakaka RT (2006). Chromatin insulators.. Annu Rev Genet.

[9] Chiu YH, Yu Q, Sandmeier JJ, Bi X (2003). A targeted histone acetyltransferase can create a sizable region of hyperacetylated chromatin and counteract the propagation of transcriptionally silent chromatin.. Genetics.

[10] Donze D, Kamakaka RT (2002). Braking the silence: how heterochromatic gene repression is stopped in its tracks.. Bioessays.

[11] Oki M, Valenzuela L, Chiba T, Ito T, Kamakaka RT (2004). Barrier proteins remodel and modify chromatin to restrict silenced domains.. Mol Cell Biol.

[12] West AG, Huang S, Gaszner M, Litt MD, Felsenfeld G (2004). Recruitment of histone modifications by USF proteins at a vertebrate barrier element.. Mol Cell.

[13] Avramova Z, Tikhonov A (1999). Are scs and scs' ‘neutral’ chromatin domain boundaries of the locus?. Trends Genet.

[14] Donze D, Kamakaka RT (2001). RNA polymerase III and RNA polymerase II promoter complexes are heterochromatin barriers in Saccharomyces cerevisiae.. Embo J.

[15] Fourel G, Boscheron C, Revardel E, Lebrun E, Hu YF (2001). An activation-independent role of transcription factors in insulator function.. EMBO Rep.

[16] Scott KC, Merrett SL, Willard HF (2006). A heterochromatin barrier partitions the fission yeast centromere into discrete chromatin domains.. Curr Biol.

[17] Huang Y, Maraia RJ (2001). Comparison of the RNA polymerase III transcription machinery in Schizosaccharomyces pombe, Saccharomyces cerevisiae and human.. Nucleic Acids Res.

[18] Hamada M, Huang Y, Lowe TM, Maraia RJ (2001). Widespread use of TATA elements in the core promoters for RNA polymerases III, II, and I in fission yeast.. Mol Cell Biol.

[19] Geiduschek EP, Kassavetis GA (2001). The RNA polymerase III transcription apparatus.. J Mol Biol.

[20] Takahashi K, Murakami S, Chikashige Y, Niwa O, Yanagida M (1991). A large number of tRNA genes are symmetrically located in fission yeast centromeres.. J Mol Biol.

[21] Takahashi K, Murakami S, Chikashige Y, Funabiki H, Niwa O (1992). A low copy number central sequence with strict symmetry and unusual chromatin structure in fission yeast centromere.. Mol Biol Cell.

[22] Noma K, Cam HP, Maraia RJ, Grewal SI (2006). A role for TFIIIC transcription factor complex in genome organization.. Cell.

[23] Hull MW, Erickson J, Johnston M, Engelke DR (1994). tRNA genes as transcriptional repressor elements.. Mol Cell Biol.

[24] Kinsey PT, Sandmeyer SB (1991). Adjacent pol II and pol III promoters: transcription of the yeast retrotransposon Ty3 and a target tRNA gene.. Nucleic Acids Res.

[25] Bolton EC, Boeke JD (2003). Transcriptional interactions between yeast tRNA genes, flanking genes and Ty elements: a genomic point of view.. Genome Res.

[26] Conesa C, Ruotolo R, Soularue P, Simms TA, Donze D (2005). Modulation of yeast genome expression in response to defective RNA polymerase III-dependent transcription.. Mol Cell Biol.

[27] Simms TA, Miller EC, Buisson NP, Jambunathan N, Donze D (2004). The Saccharomyces cerevisiae TRT2 tRNAThr gene upstream of STE6 is a barrier to repression in MATalpha cells and exerts a potential tRNA position effect in MATa cells.. Nucleic Acids Res.

[28] Djupedal I, Portoso M, Spahr H, Bonilla C, Gustafsson CM (2005). RNA Pol II subunit Rpb7 promotes centromeric transcription and RNAi-directed chromatin silencing.. Genes Dev.

[29] Cam HP, Sugiyama T, Chen ES, Chen X, FitzGerald PC (2005). Comprehensive analysis of heterochromatin- and RNAi-mediated epigenetic control of the fission yeast genome.. Nat Genet.

[30] Rea S, Eisenhaber F, O'Carroll D, Strahl BD, Sun ZW (2000). Regulation of chromatin structure by site-specific histone H3 methyltransferases.. Nature.

[31] Hall IM, Shankaranarayana GD, Noma K, Ayoub N, Cohen A (2002). Establishment and maintenance of a heterochromatin domain.. Science.

[32] Nakayama J, Rice JC, Strahl BD, Allis CD, Grewal SI (2001). Role of histone H3 lysine 9 methylation in epigenetic control of heterochromatin assembly.. Science.

[33] Kimura A, Horikoshi M (2004). Partition of distinct chromosomal regions: negotiable border and fixed border.. Genes Cells.

[34] Gordon M, Holt D, Panigrahi A, Wilhelm BT, Erdjument-Bromage H (2007). Genome-wide dynamics of SAPHIRE, an essential complex for gene activation and chromatin boundaries.. Mol Cell Biol.

[35] Chung JH, Whiteley M, Felsenfeld G (1993). A 5′ element of the chicken beta-globin domain serves as an insulator in human erythroid cells and protects against position effect in Drosophila.. Cell.

[36] Litt MD, Simpson M, Recillas-Targa F, Prioleau MN, Felsenfeld G (2001). Transitions in histone acetylation reveal boundaries of three separately regulated neighboring loci.. Embo J.

[37] Oki M, Kamakaka RT (2005). Barrier function at HMR.. Mol Cell.

[38] Chedin S, Ferri ML, Peyroche G, Andrau JC, Jourdain S (1998). The yeast RNA polymerase III transcription machinery: a paradigm for eukaryotic gene activation.. Cold Spring Harb Symp Quant Biol.

[39] Chedin S, Riva M, Schultz P, Sentenac A, Carles C (1998). The RNA cleavage activity of RNA polymerase III is mediated by an essential TFIIS-like subunit and is important for transcription termination.. Genes Dev.

[40] Kassavetis GA, Kumar A, Letts GA, Geiduschek EP (1998). A post-recruitment function for the RNA polymerase III transcription-initiation factor IIIB.. Proc Natl Acad Sci U S A.

[41] Wittig S, Wittig B (1982). Function of a tRNA gene promoter depends on nucleosome position.. Nature.

[42] Morse RH, Roth SY, Simpson RT (1992). A transcriptionally active tRNA gene interferes with nucleosome positioning in vivo.. Mol Cell Biol.

[43] Gerlach VL, Whitehall SK, Geiduschek EP, Brow DA (1995). TFIIIB placement on a yeast U6 RNA gene in vivo is directed primarily by TFIIIC rather than by sequence-specific DNA contacts.. Mol Cell Biol.

[44] Lan F, Zaratiegui M, Villen J, Vaughn MW, Verdel A (2007). S. pombe LSD1 Homologs Regulate Heterochromatin Propagation and Euchromatic Gene Transcription.. Mol Cell.

[45] Thompson M, Haeusler RA, Good PD, Engelke DR (2003). Nucleolar clustering of dispersed tRNA genes.. Science.

[46] Wang L, Haeusler RA, Good PD, Thompson M, Nagar S (2005). Silencing near tRNA genes requires nucleolar localization.. J Biol Chem.

[47] Donze D, Adams CR, Rine J, Kamakaka RT (1999). The boundaries of the silenced HMR domain in Saccharomyces cerevisiae.. Genes Dev.

[48] Braglia P, Dugas SL, Donze D, Dieci G (2007). Requirement of Nhp6 proteins for transcription of a subset of tRNA genes and heterochromatin barrier function in Saccharomyces cerevisiae.. Mol Cell Biol.

[49] Pidoux AL, Allshire RC (2004). Kinetochore and heterochromatin domains of the fission yeast centromere.. Chromosome Res.

[50] Ekwall K, Javerzat JP, Lorentz A, Schmidt H, Cranston G (1995). The chromodomain protein Swi6: a key component at fission yeast centromeres.. Science.

[51] Ekwall K, Nimmo ER, Javerzat JP, Borgstrom B, Egel R (1996). Mutations in the fission yeast silencing factors clr4+ and rik1+ disrupt the localisation of the chromo domain protein Swi6p and impair centromere function.. J Cell Sci.

[52] Moreno S, Klar A, Nurse P (1991). Molecular genetic analysis of fission yeast Schizosaccharomyces pombe.. Methods Enzymol.

